# Supplementary figures and images for: Functional constraints of wtf killer meiotic drivers
Source: PLoS Genet. 2025 Feb 18;21(2):e1011534. doi: 10.1371/journal.pgen.1011534 (PMC11892871; doi:10.1371/journal.pgen.1011534)

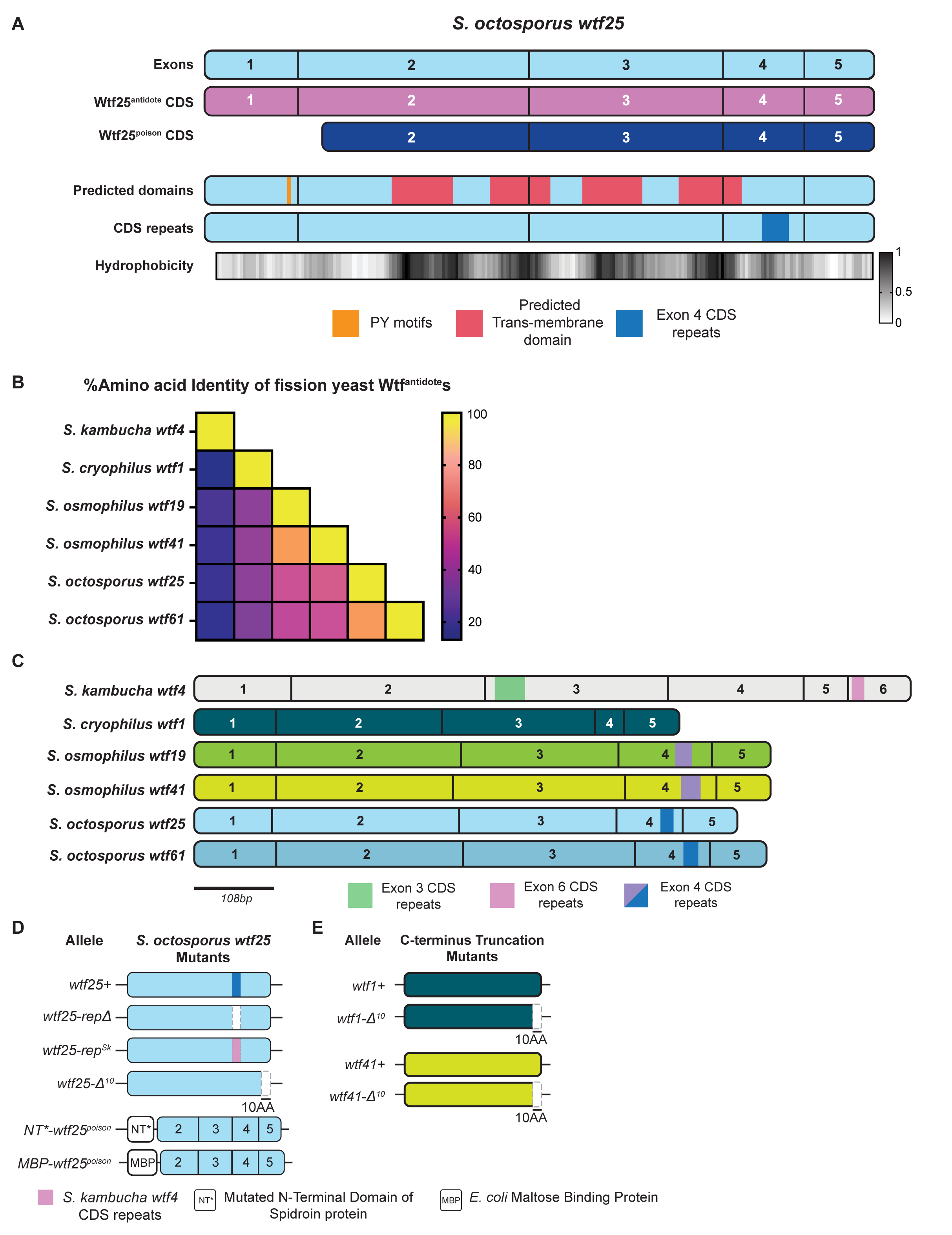

Supplement: S1 Fig — A. A cartoon of S. octosporus wtf25 coding sequence (CDS). Wtf25antidote coding sequence is shown in purple, which includes exons 1–5. The Wtf4poison coding sequence is shown in navy, which begins at the 27th base pair of exon 2 and extends through exon 5. Row 4 depicts the predicted transmembrane domains (in red) and PY motif (in mustard). Row 5 depicts the CDS repeats found in exon 4 (in cobalt). Row 6 depicts the normalized hydrophobicity of Wtf25 proteins from ProtScale, with the Kyle and Doolittle Hydropathy scale [23]. The higher the number on the scale, the higher the hydrophobicity of the amino acid. See S2 and S3 Tables for more detailed descriptions. B. Pairwise amino acid identity of the 6 Wtfantidote proteins shown. The amino acid sequences were aligned using Geneious Prime (2023.0.4), and the percentage amino acid identity is depicted as a heatmap, with yellow being 100% identity. C. Depiction of CDS repeats and lengths of 6 wtfantidote CDSs. The CDS repeats found in exon 6 of S. kambucha wtf4 are homologous to those found in exon 4 of the other wtf genes [19]. The scale bar represents 108 base pairs (bp). D-E. S. octosporus wtf25 (D), S. cryophilus wtf1, and S. osmophilus wtf41 (E) mutants constructed in this study. The categories have their respective wild type allele shown on top. See S1 Table for a comprehensive overview of the alleles and their phenotypes. (TIF) [file pgen.1011534.s001.tif]

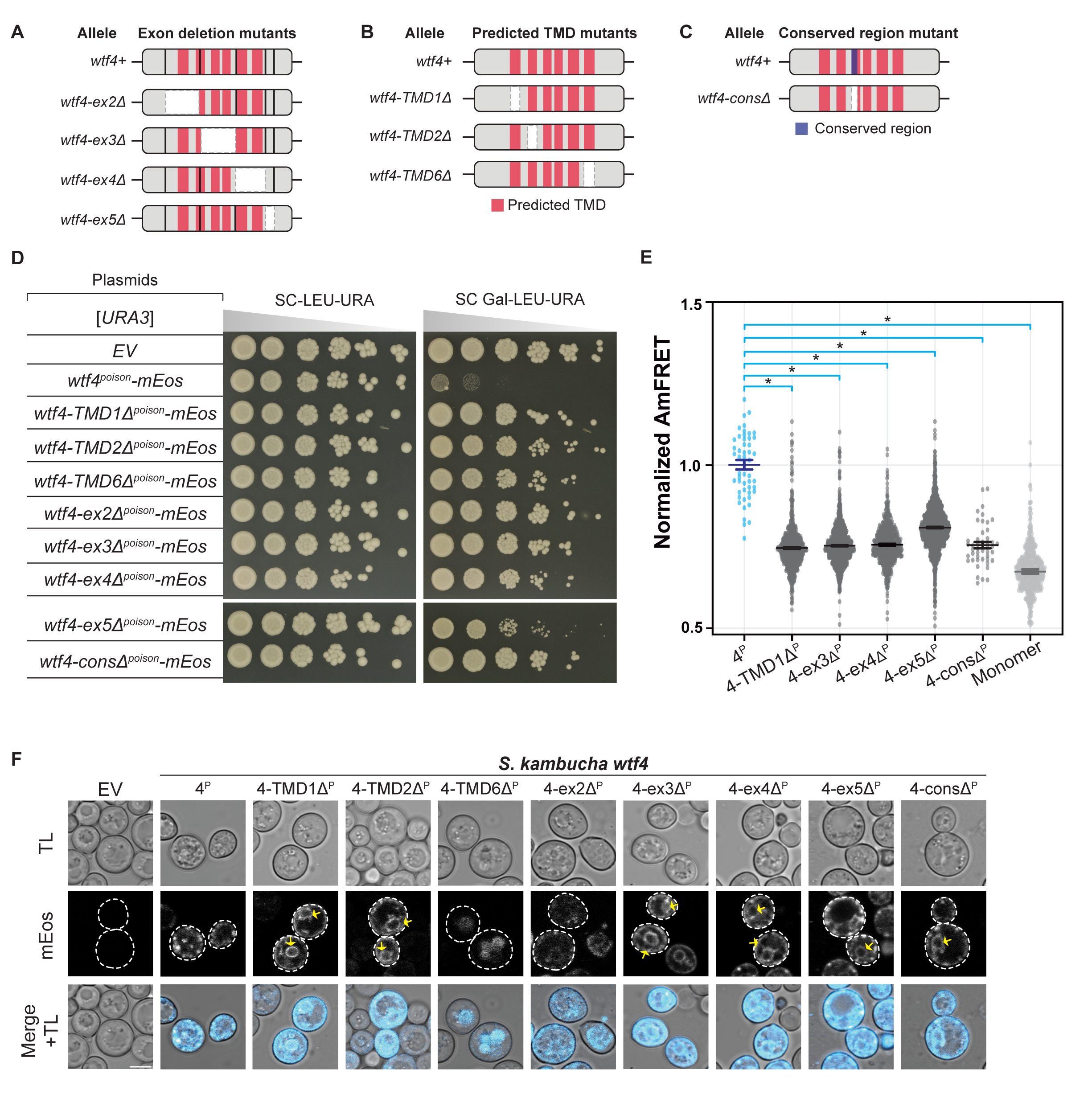

Supplement: S2 Fig — Cartoon of S. kambucha wtf4 exon deletion mutants (A), predicted transmembrane domain (TMD) deletion mutants (B) and a mutant that deletes a 9 amino acid conserved region encoded in exon 3 (C). D. A spot assay of cells serially diluted and plated on SC-LEU-URA and SC Gal-LEU-URA plates and grown at 30 ℃ for 3 days. Each strain carries an empty [LEU2] plasmid, and either an empty [URA3] plasmid (EV) or the indicated wtf4poison-mEos alleles under the control of a galactose-inducible promoter. The horizontal break in the image for each plate is due to rearrangement of the image to facilitate easy comparison. All strains were grown on the same plates (i.e., one SC-LEU-URA or SC Gal-LEU-URA plate). E. AmFRET values for three technical replicates of the specified Wtf4poison-mEos proteins and monomer-mEos (negative control). The median is indicated with a solid line and the bars represent the interquartile range. For easier comparison, the values were normalized so that Wtf4poison had a median of 1 in each experiment. The data shown here do not include outliers. See S2 Data for the complete dataset and p-values. Statistical significance: *p<0.008, **p<0.0008, t-tests between the means of each replicate, with Bonferroni correction. F. Representative images of the same strains depicted in D were induced in galactose media for 4 hours at 30 ℃ to express the indicated mEos-tagged proteins. The images are not at the same brightness and contrast settings to clearly show localization of tagged proteins. Yellow arrows indicate endoplasmic reticulum-like localization. 4P indicates Wtf4poison, TL is transmitted light, and the scale bar is 4 µm. (TIF) [file pgen.1011534.s002.tif]

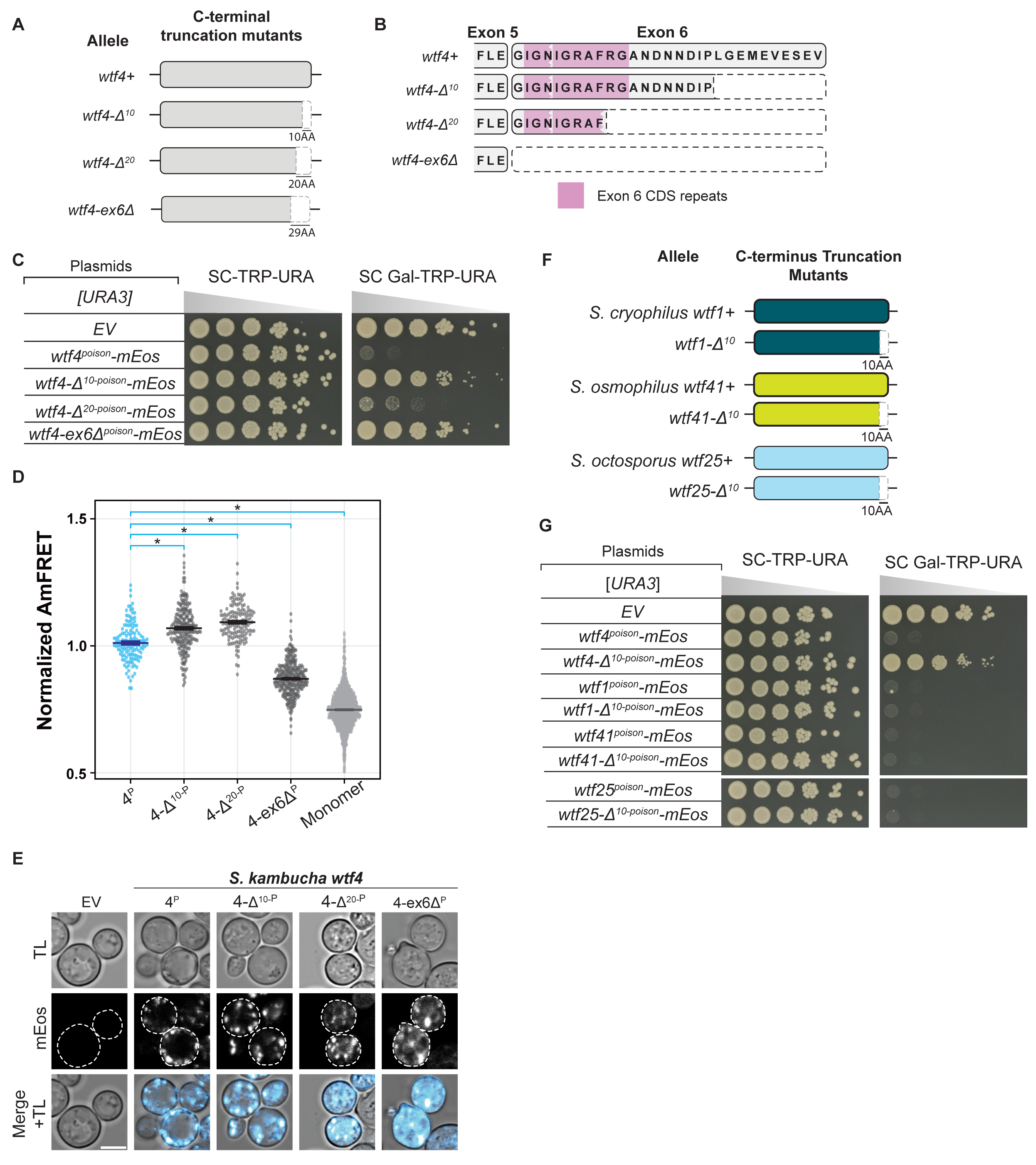

Supplement: S3 Fig — A. Cartoon of C-terminal truncation mutants of S. kambucha wtf4. B. Cartoon illustrating the amino acids lost with the C-terminal truncation alleles of wtf4. The amino acids comprising the exon 6 coding sequence repeats are highlighted in pink. C. A spot assay of cells serially diluted and plated on SC-TRP-URA and SC Gal-TRP-URA plates and grown at 30 ℃ for 3 days. Each strain carries both an empty [TRP1] plasmid (EV) and a [URA3] plasmid that is either empty or carries the indicated wtf4poison-mEos allele under the control of a galactose inducible promoter. D. AmFRET values for three technical replicates of the specified Wtf4poison-mEos alleles and monomer-mEos (negative control). The median is indicated with a solid line and the bars represent the interquartile range. For easier comparison, the values were normalized so that Wtf4poison had a median of 1 in each experiment. The data shown here do not include outliers. See S2 Data for the complete dataset and p-values. Statistical significance: *p<0.0125, t-test with Bonferroni correction. E. Representative images of the same strains depicted in C were induced with galactose media for 4 hours at 30 ℃ to express the indicated mEos-tagged proteins. The images are not at the same brightness and contrast settings to clearly show localization of tagged proteins. 4P indicates Wtf4poison, TL is transmitted light, and the scale bar is 4 µm. F. Cartoons of C-terminal truncation mutants of S. cryophilus wtf1 (wtf1-Δ10), S. osmophilus wtf41 (wtf41-Δ10), and S. octosporus wtf25 (wtf25-Δ10). G. Spot assays of cells serially diluted and plated on SC-TRP-URA and SC Gal-TRP-URA plates and grown at 30 ℃ for 3 days. Each strain carries both an empty [TRP1] plasmid (EV) and an empty [URA3] plasmid (EV), or the indicated wtfpoison-mEos allele under the control of a galactose-inducible promoter. The horizontal breaks in the images for each plate are due to rearrangements of the image to facilitate easy comparison. All strains were grown [file pgen.1011534.s003.tif]

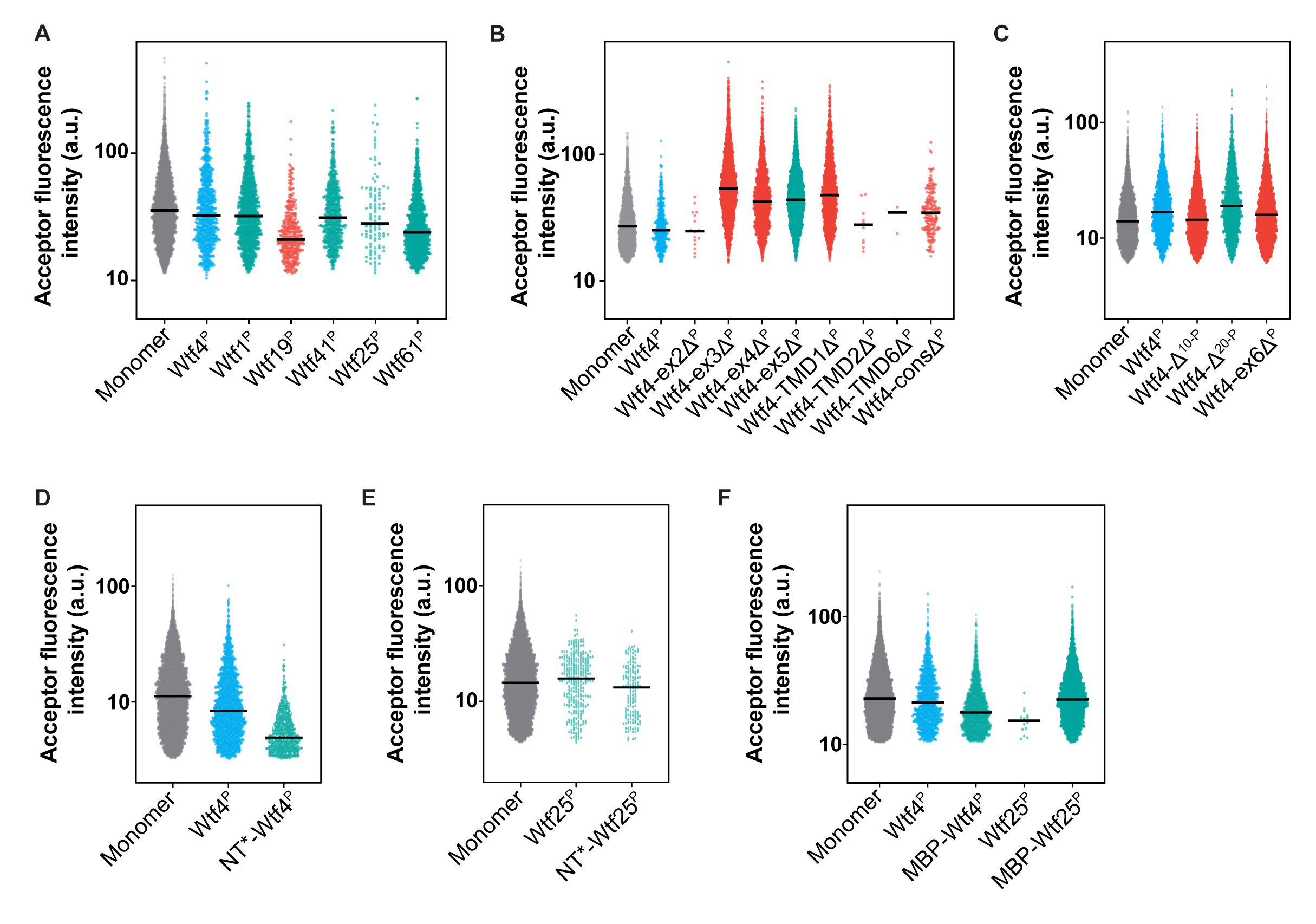

Supplement: S4 Fig — A-F Acceptor fluorescence intensity (in arbitrary units, a.u.) of similarly sized live cells across all Wtf DAmFRET experiments in this study, with the line indicating the median of the population. Data represented here is from the following experiments: A corresponds to Fig 2B, B corresponds to S2E Fig, C corresponds to S3D Fig, D corresponds to Fig 4C, E corresponds alleles in S6 Fig, and F corresponds to S7D Fig. The Y-axis is scaled to a log10 scale. The data are color coded, with toxic poisons in teal, and non-toxic alleles in orange. (TIF) [file pgen.1011534.s004.tif]

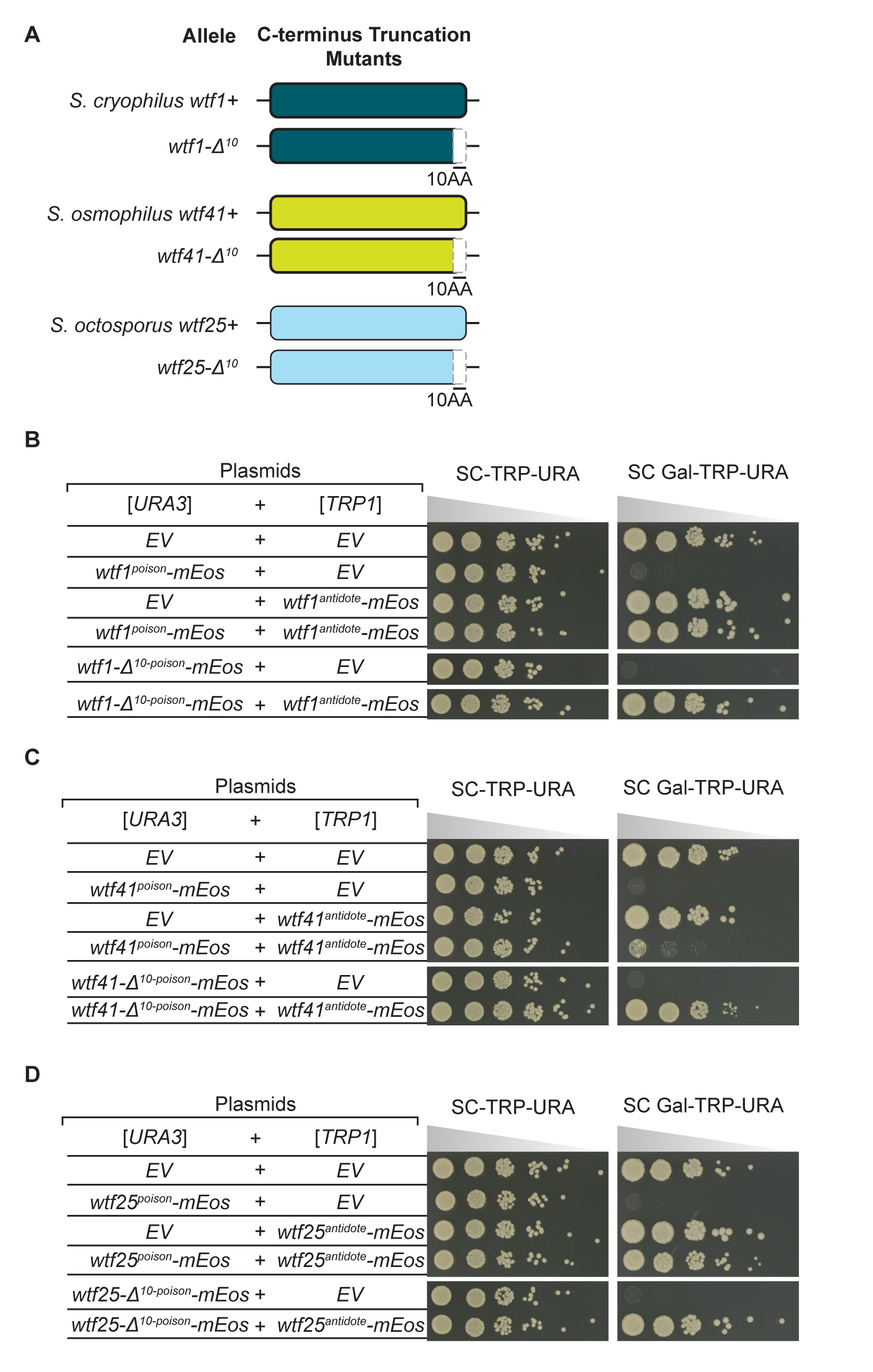

Supplement: S5 Fig — A. Cartoon of S. cryophilus wtf1, S. osmophilus wtf41, and S. octosporus wtf25 C-terminal truncation mutants. B-D. Spot assays of cells serially diluted on SC-TRP-URA and SC Gal-TRP-URA plates and grown at 30 ℃ for 3 days. Each strain carries both a [URA3] and a [TRP1] plasmid. The plasmids are either empty (EV) or carry the indicated wtf alleles under the control of galactose-inducible promoters. The horizontal breaks in the images within a panel are due to rearrangements of the images to facilitate easy comparison. All strains within a panel were grown on the same plates (i.e., one SC-TRP-URA or SC Gal-TRP-URA plate). (TIF) [file pgen.1011534.s005.tif]

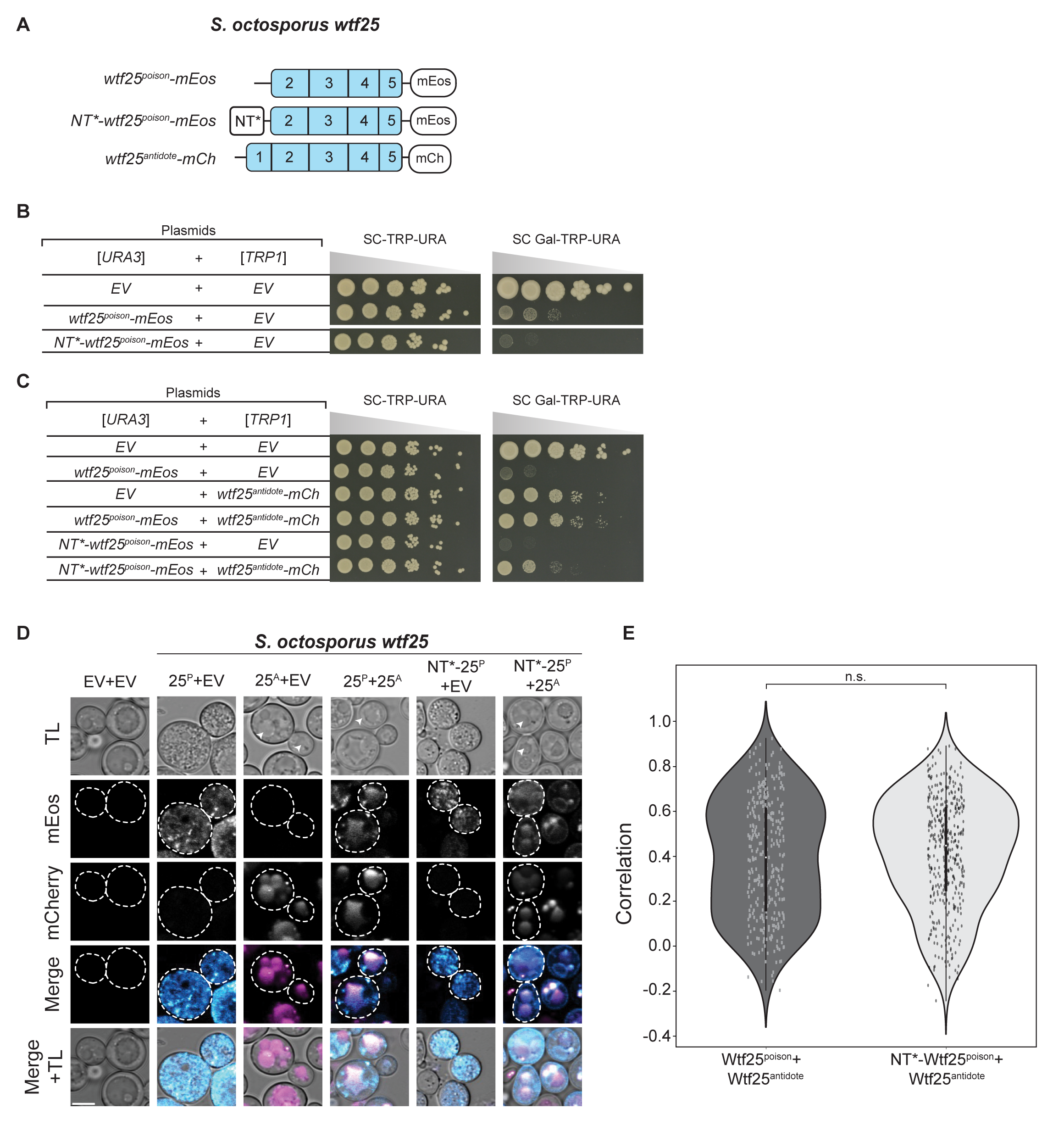

Supplement: S6 Fig — A. Cartoon of alleles used in this experiment (B-E). The NT* tag has a general anti-aggregation property [32]. B-C. Spot assays of cells serially diluted on SC-TRP-URA and SC Gal-TRP-URA plates and grown at 30 ℃ for 4 days. Each strain carries both a [URA3] and a [TRP1] plasmid. The plasmids are either empty (EV) or carry the indicated wtf25 alleles under the control of galactose-inducible promoters. In B, the horizontal break in the image of each plate is due to a rearrangement of the image to facilitate easy comparison. All strains within a panel were grown on the same plates (i.e., one SC-TRP-URA or SC Gal-TRP-URA plate for panel B). D. Representative images of the same strains shown in C were induced with galactose media for 4 hours at 30 ℃ to express the indicated Wtf25poison-mEos proteins and/or Wtf25antidote-mCherry. The images are not at the same brightness and contrast settings to clearly show localization of tagged proteins. 25P indicates Wtf25poison, 25A indicates Wtf25antidote, TL is transmitted light, and the scale bar is 4 µm. E. Pearson’s Correlation between mCherry and mEos signal in cells from D expressing the specified proteins. N>100, p>0.05, t-test. (TIF) [file pgen.1011534.s006.tif]

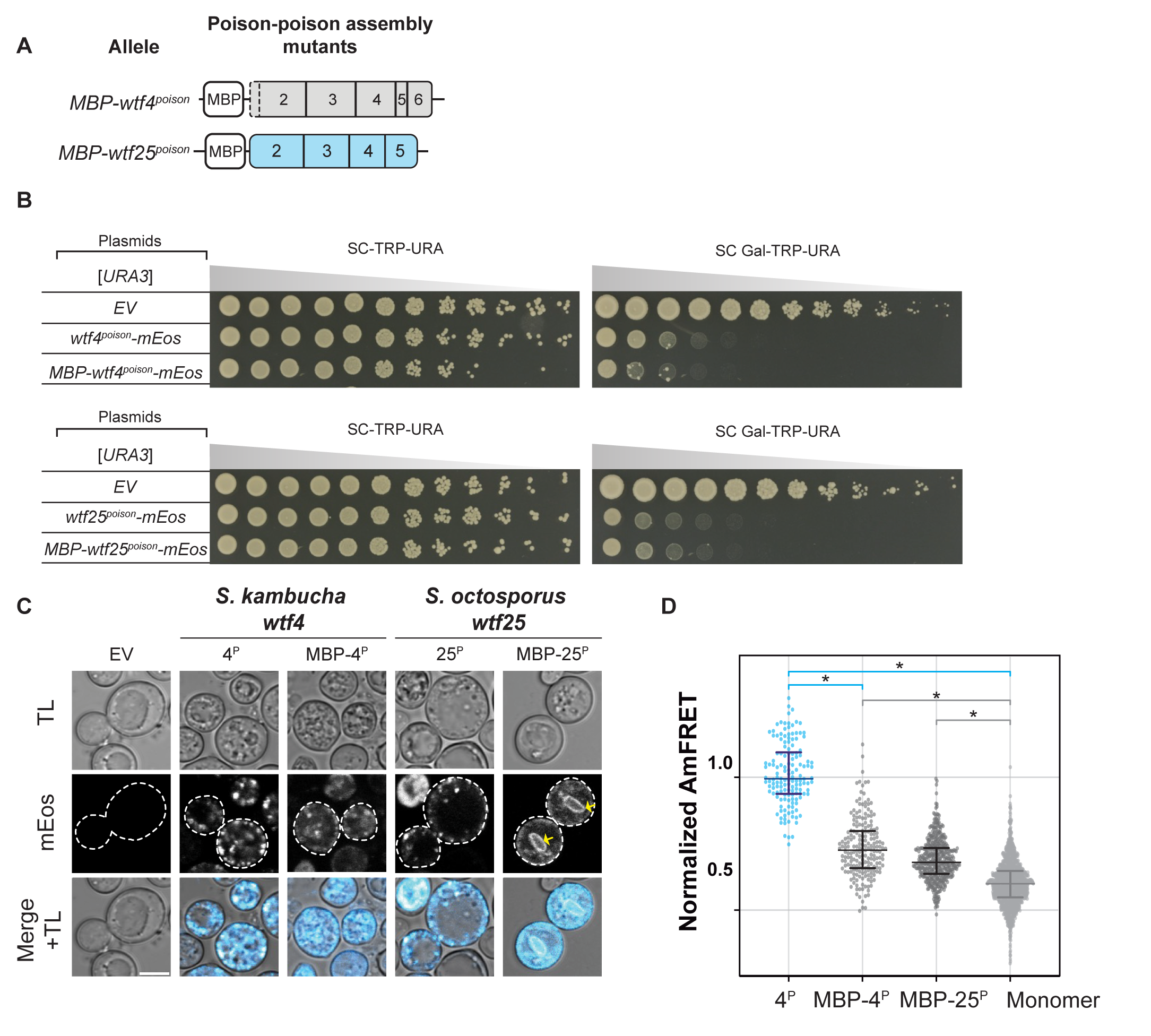

Supplement: S7 Fig — A. Cartoon of alleles used in this experiment (B-C). MBP is the E. coli Maltose Binding Protein [76]. B. Spot assay of cells serially diluted and plated on SC-TRP-URA and SC Gal-TRP-URA plates and grown at 30 ℃ for 4 days. Each strain carries both an empty [TRP1] plasmid and a [URA3] plasmid that is either empty (EV) or carries the indicated wtfpoison-mEos allele under the control of a galactose-inducible promoter. The horizontal break in the image of each plate is due to rearrangement of the image to facilitate easy comparison. All strains were grown on the same plates (i.e., one SC Gal-TRP-URA plate or SC-Trp-Ura). C. Representative images of the same strains depicted in B induced with galactose media for 4 hours at 30 ℃ to express the indicated mEos-tagged proteins. The images are not at the same brightness and contrast settings to clearly show localization of tagged proteins. Yellow arrows indicate endoplasmic reticulum-like localization. 4P indicates Wtf4poison, 25P indicates Wtf25poison, TL is transmitted light, and the scale bar is 4 µm. D. AmFRET values for three technical replicates of the specified Wtfpoison-mEos alleles and monomer-mEos (negative control). The median is indicated with a solid line and the bars represent the interquartile range. For easier comparison, the values were normalized so that Wtf4poison had a median of 1 in each experiment. The data shown do not include outliers. See S2 Data for the complete dataset and p-values. Statistical significance: *p<0.016, t-test with Bonferroni correction. (TIF) [file pgen.1011534.s007.tif]

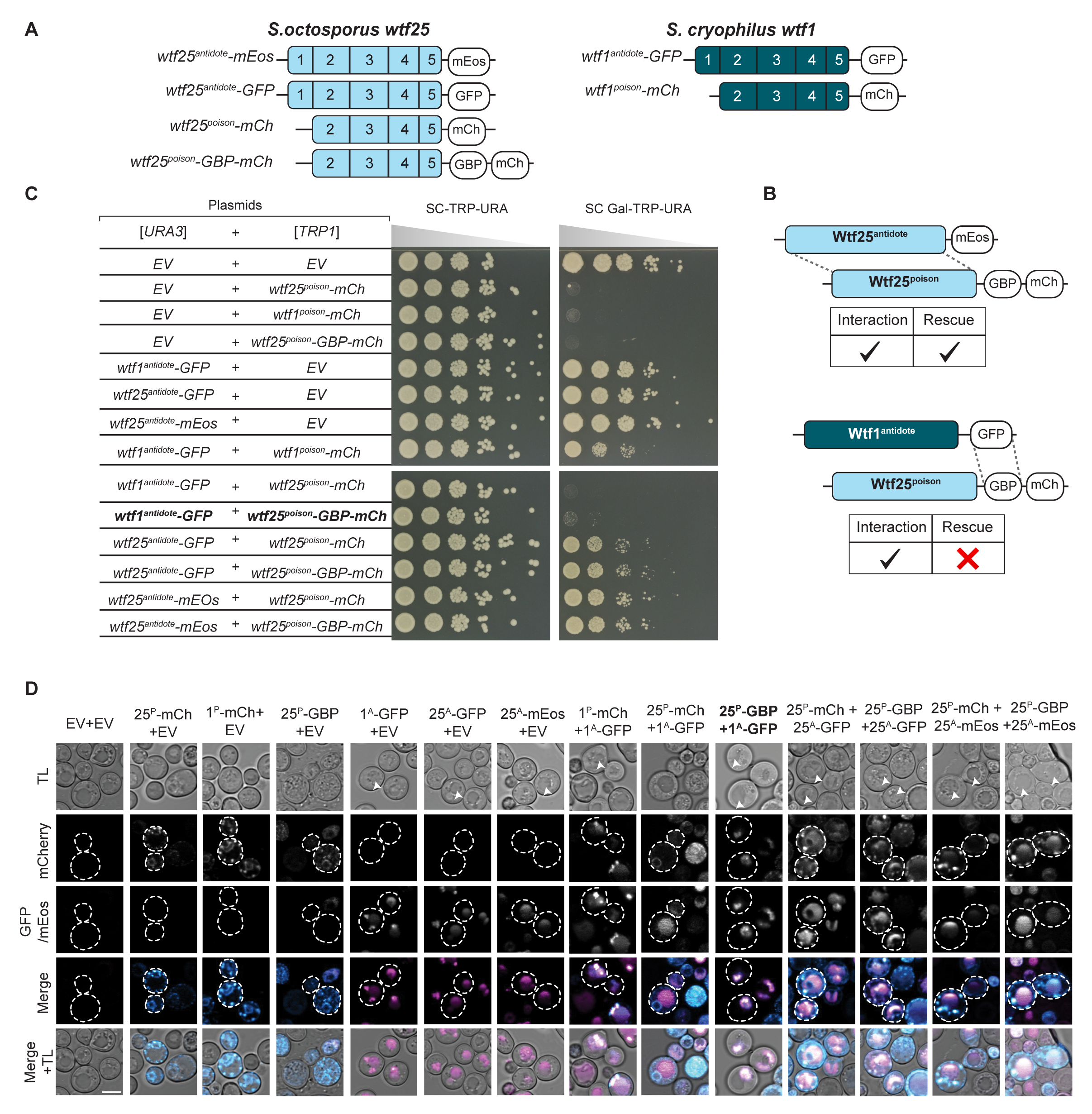

Supplement: S8 Fig — A. Cartoon of wtf4antidote exon 1 and mutants that relocate, or both relocate and mutate the exon. B. AmFRET values for three technical replicates of the specified mEos-tagged proteins and monomer-mEos (negative control). The median is indicated with a solid line and the bars represent 1.5 times the interquartile range. For easier comparison, the values were normalized so that Wtf4poison had a median of 1 in each experiment. The data shown do not include outliers. See S2 Data for the complete dataset and p-values. Statistical significance: *p<0.0125, t-tests with Bonferroni correction. C-D. Spot assays of cells serially diluted on SC-TRP-URA and SC Gal-TRP-URA plates and grown at 30 ℃ for 3 days. Each strain carries both a [URA3] and a [TRP1] plasmid. The plasmids are either empty (EV) or carry the indicated wtf4 alleles under the control of galactose-inducible promoters. The horizontal breaks in the images of each plate in panels C and D are due to rearrangements of the images to facilitate easy comparison. All strains within a panel were grown on the same plates (i.e., one SC-TRP-URA or SC Gal-TRP-URA plate for panel C). E. Representative images of the same strains shown in C induced with galactose media for 4 hours at 30 ℃ to express the Wtf4poison-mEos, Wtf4 exon1-mCherry, or both proteins. F. Representative images of the same strains shown in D were induced with galactose media for 4 hours at 30 ℃ to the indicated wtf4 alleles. In E-F, arrows in the transmitted light images indicate vacuoles. The images are not at the same brightness and contrast settings to clearly show localization of the tagged proteins. 4P indicates Wtf4poison, 4A indicates Wtf4antidote, Ex1 is Wtf4 exon1, TL is transmitted light, and the scale bar is 4 µm. (TIF) [file pgen.1011534.s008.tif]

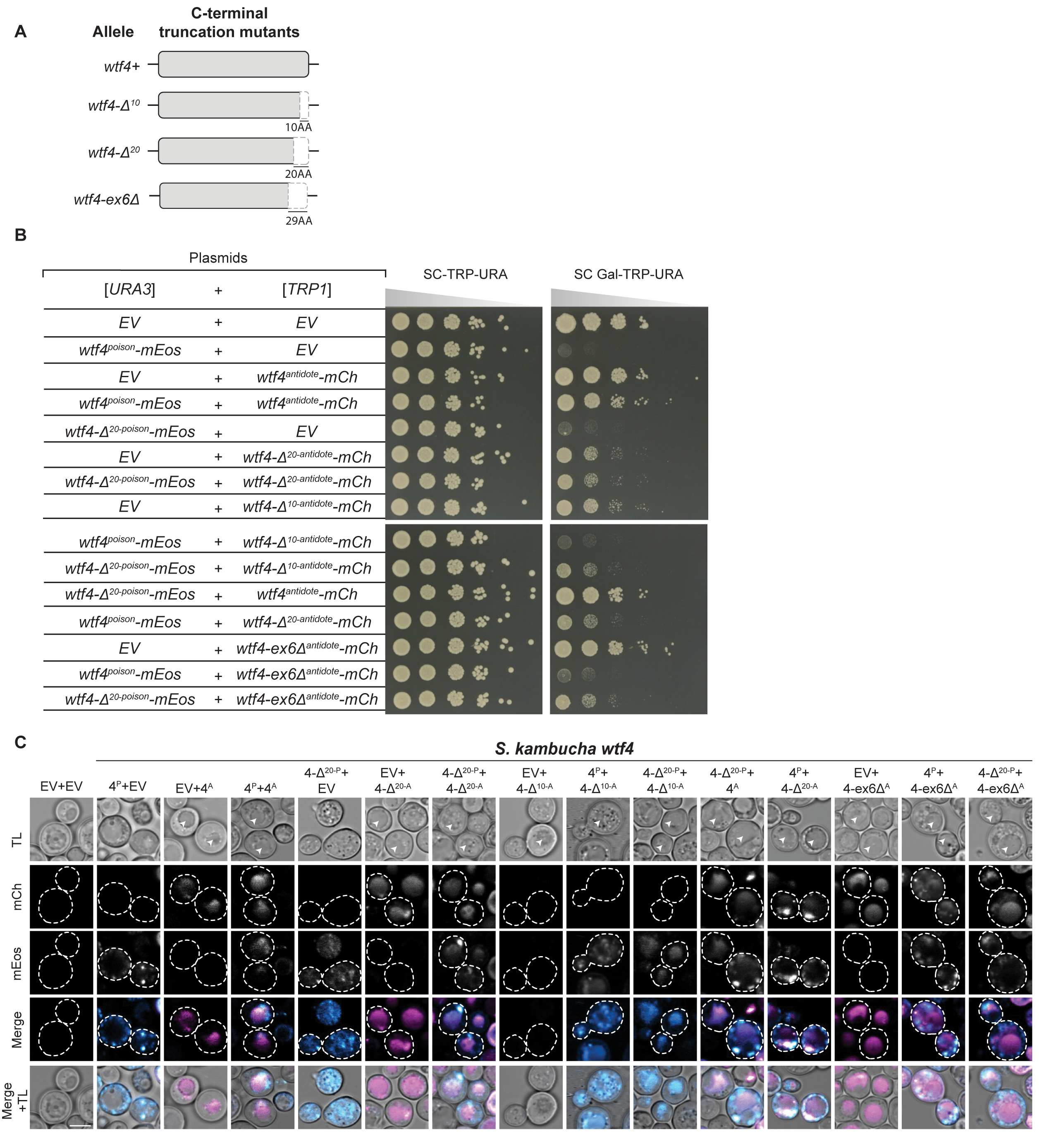

Supplement: S10 Fig — A. Cartoon of S. kambucha wtf4 C-terminal truncation mutants. B. Spot assay of cells serially diluted and plated on SC-TRP-URA and SC Gal-TRP-URA plates and grown at 30 ℃ for 3 days. Each strain carries both a [URA3] and a [TRP1] plasmid. The plasmids are either empty (EV) or carry the indicated wtf4 alleles under the control of galactose-inducible promoters. The horizontal breaks in the images are due to rearrangements of the images to facilitate easy comparison. All strains within a panel were grown on the same plates (i.e., one SC-TRP-URA or SC Gal-TRP-URA plate). C. Representative images the strains depicted in B were induced in galactose for 4 hours at 30 ℃ to express the indicated Wtf4 proteins. The images are not at the same brightness and contrast settings to clearly show localization of tagged proteins. The arrows in the TL panels highlight vacuoles. 4P indicates Wtf4poison, 4A indicates Wtf4antidote, TL indicates transmitted light, and the scale bar is 4 µm. (TIF) [file pgen.1011534.s010.tif]

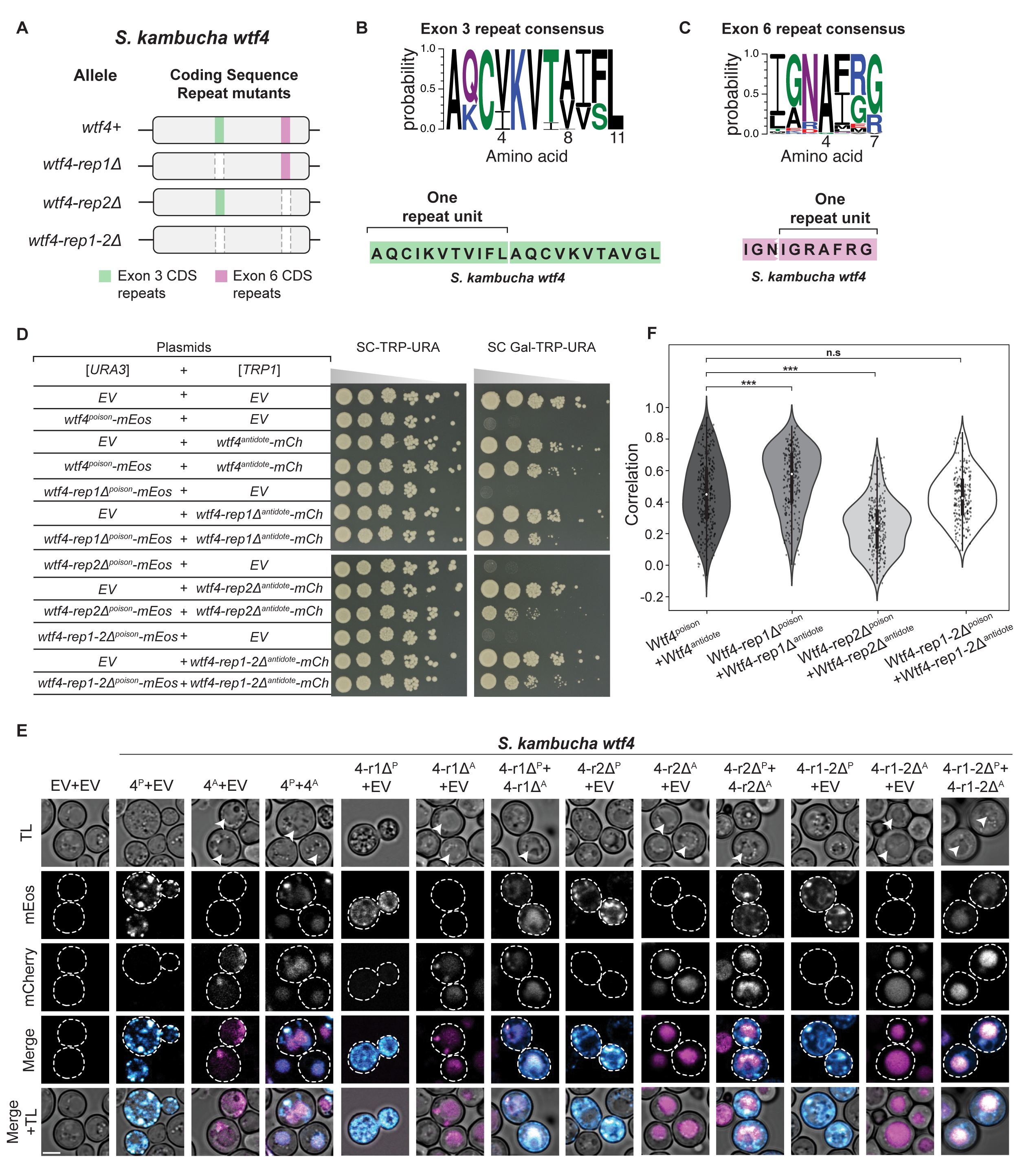

Supplement: S11 Fig — A. Cartoon of exon 3 and exon 6 coding sequence repeat deletion mutants of S. kambucha wtf4. B-C. Logo for amino acids encoded by the repeats found in exon 3 (B) and exon 6 (C) of S. pombe wtf genes from [20]. The sequence of the amino acids encoded in each repeat region in S. kambucha wtf4 is shown below each logo. D. Spot assay of cells serially diluted and plated on SC-TRP-URA and SC Gal-TRP-URA plates and grown at 30 ℃ for 3 days. Each strain carries both a [URA3] and a [TRP1] plasmid. The plasmids are either empty (EV) or carry the indicated wtf4 alleles under the control of galactose-inducible promoters. The horizontal break in the image of each plate is due to rearrangements of the images to facilitate easy comparison. All strains within a panel were grown on the same plates (i.e., one SC-TRP-URA or SC Gal-TRP-URA plate). E. Representative images of the same strains as depicted in D were induced in galactose for 4 hours at 30 ℃ to express the indicated Wtf4 proteins. The images are not at the same brightness and contrast settings to clearly show localization of tagged proteins. The arrows in the TL panels highlight vacuoles. 4P indicates Wtf4poison, 4A indicates Wtf4antidote, TL indicates transmitted light, and the scale bar is 4 µm. F. Pearson’s Correlation between mEos and mCherry signal in cells expressing the specified constructs from E. N>100, ***p<0.001, t-test. (TIF) [file pgen.1011534.s011.tif]

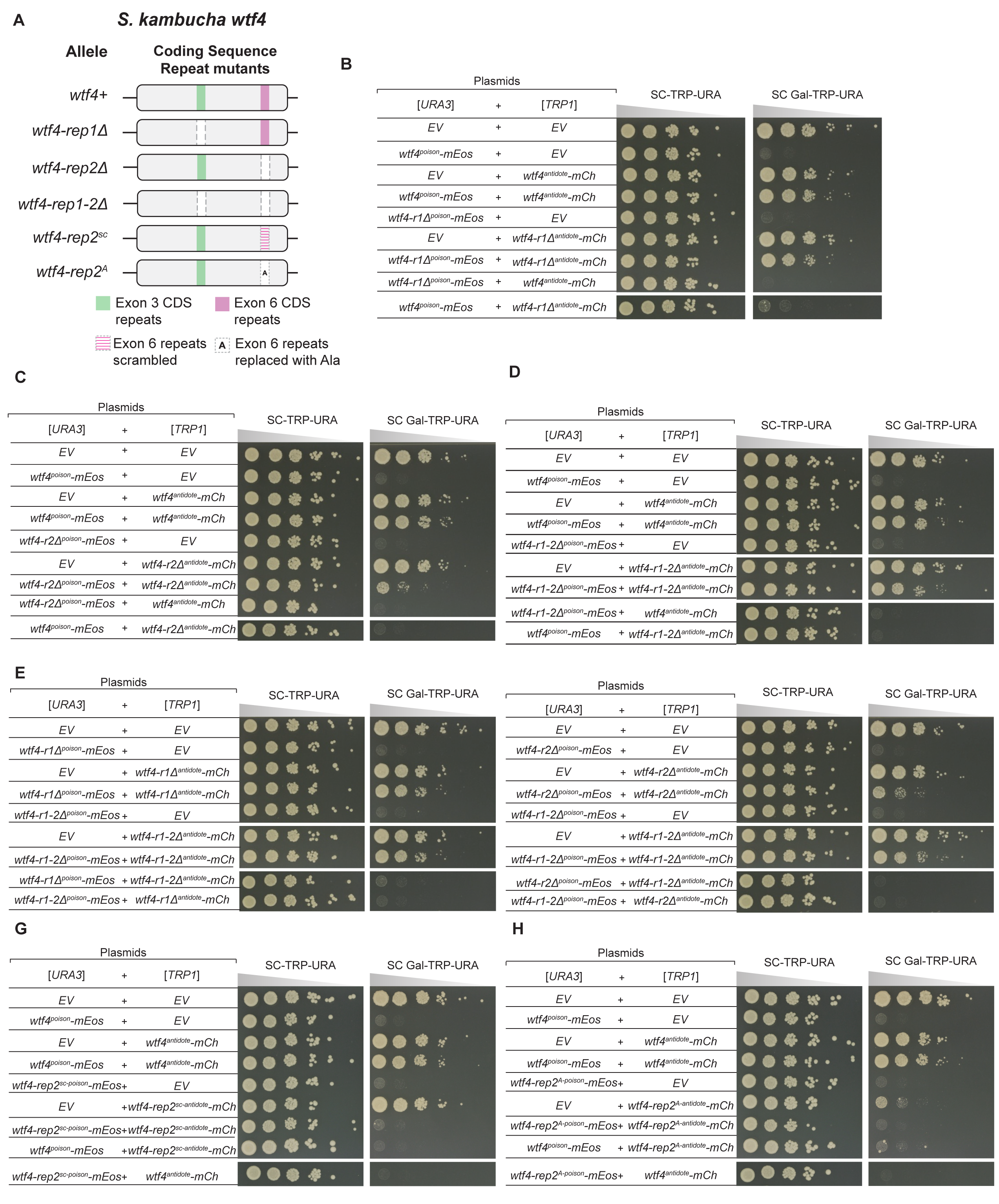

Supplement: S12 Fig — A. Cartoon of coding sequence repeat mutants of S. kambucha wtf4. B-H. Spot assays of cells serially diluted on SC-TRP-URA and SC Gal-TRP-URA plates and grown at 30 ℃ for 3 days. Each strain carries both a [URA3] and a [TRP1] plasmid. The plasmids are either empty (EV) or carry the indicated wtf4 alleles under the control of galactose-inducible promoters. The horizontal breaks in the images in within a panel are due to rearrangements of the images to facilitate easy comparison. All strains within a panel were grown on the same plates (i.e., one SC-TRP-URA or SC Gal-TRP-URA plate in panel B). (TIF) [file pgen.1011534.s012.tif]

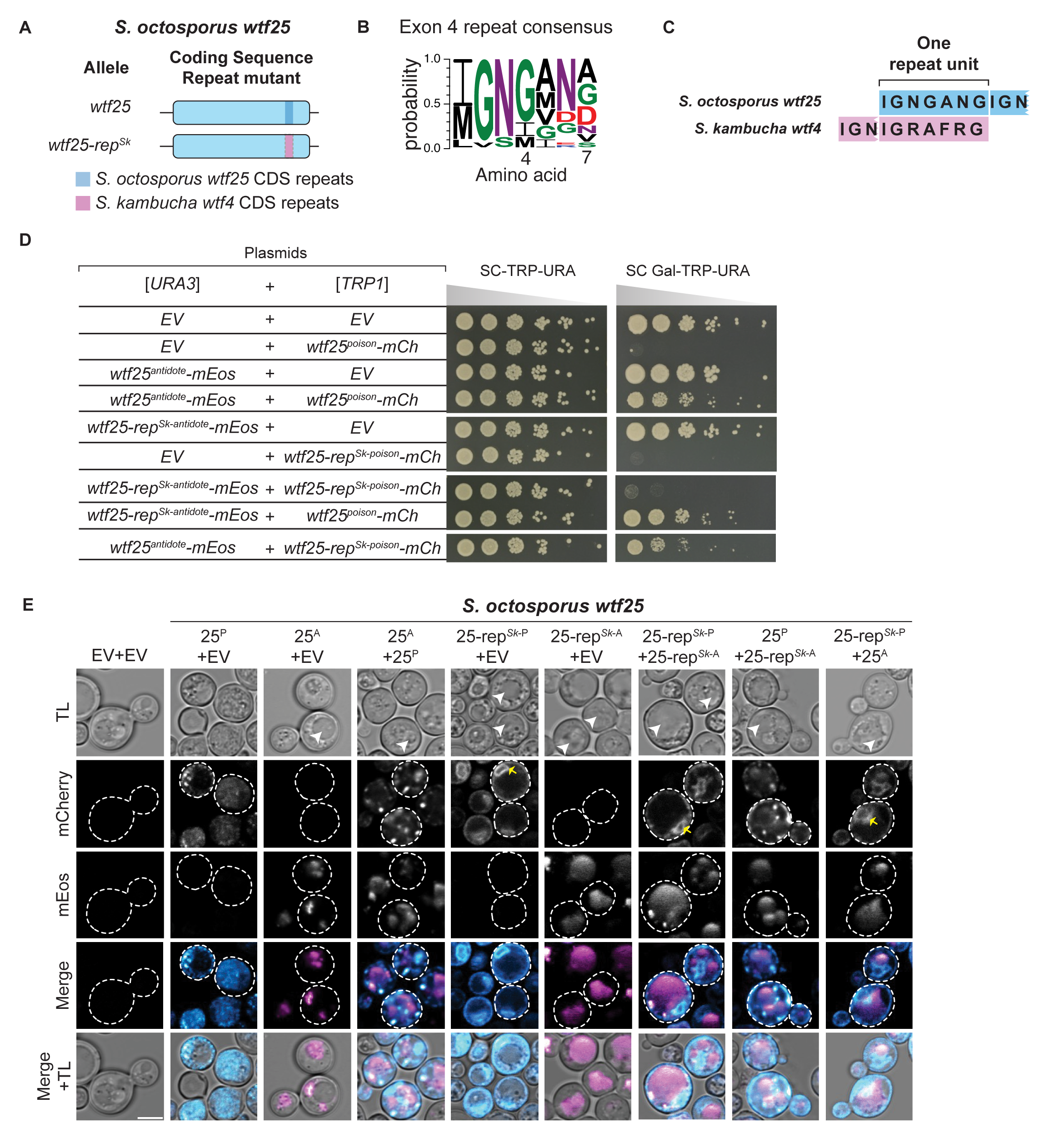

Supplement: S13 Fig — A. Cartoon of a coding sequence repeat mutant of S. octosporus wtf25. B. Logo representing the amino acids encoded by the repeats found in exon 4 of S. octosporus wtf genes from [19]. C. The amino acids encoded by the exon 4 repeats of S. octosporus wtf25 and by the exon 6 repeats of S. kambucha wtf4. D. Spot assay of cells serially diluted and plated on SC-TRP-URA and SC Gal-TRP-URA plates and grown at 30 ℃ for 3 days. Each strain carries both a [URA3] and a [TRP1] plasmid. The plasmids are either empty (EV) or carry the indicated wtf25 alleles under the control of galactose-inducible promoters. The horizontal breaks in the images are due to rearrangements of the images to facilitate easy comparison. All strains within a panel were grown on the same plates (i.e., one SC-TRP-URA or SC Gal-TRP-URA plate). E. Representative images of the same strains depicted in D were induced in galactose for 4 hours at 30 ℃ to express the indicated Wtf25 proteins. The images are not at the same brightness and contrast settings to clearly show localization of tagged proteins. The arrows in the TL panel highlight vacuoles. Yellow arrows indicate endoplasmic reticulum-like localization. 25P indicates Wtf25poison, 25A indicates Wtf25antidote, TL indicates transmitted light, and the scale bar is 4 µm. (TIF) [file pgen.1011534.s013.tif]

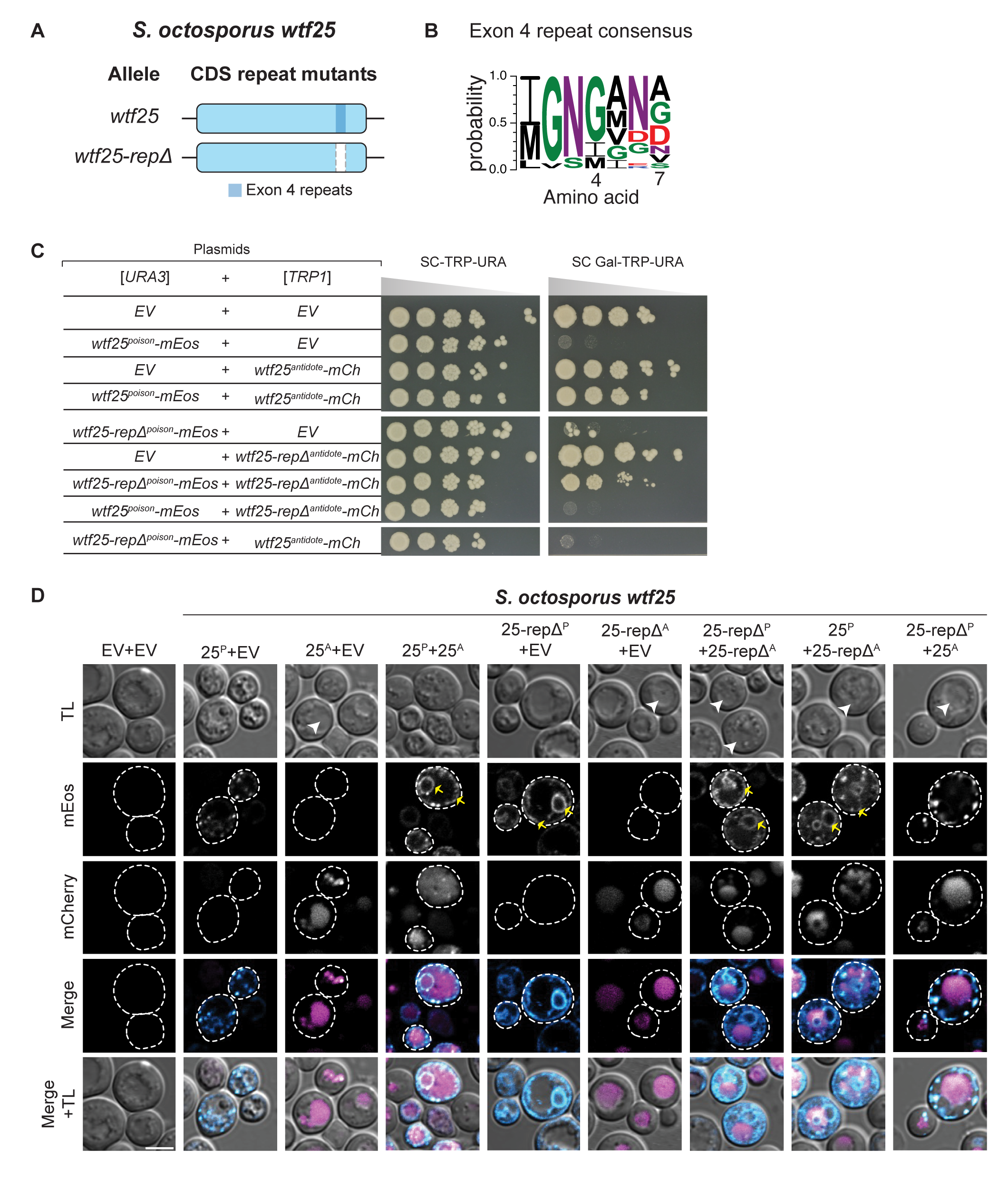

Supplement: S14 Fig — A. Cartoon of S. octosporus wtf25 exon 4 coding sequence repeat deletion mutant. B. Logo for the amino acids encoded by the repeats found in exon 4 of S. octosporus wtf genes from [19] C. Spot assay of cells serially diluted and plated on SC-TRP-URA and SC Gal-TRP-URA plates and grown at 30 ℃ for 4 days. Each strain carries both a [URA3] and a [TRP1] plasmid. The plasmids are either empty (EV) or carry the indicated wtf25 alleles under the control of galactose-inducible promoters. The horizontal breaks in the images are due to rearrangements of the images to facilitate easy comparison. All strains within a panel were grown on the same plates (i.e., one SC-TRP-URA or SC Gal-TRP-URA plate). D. Representative images the same strains depicted in C were induced in galactose for 4 hours at 30 ℃ to express the indicated Wtf25 proteins. The images are not at the same brightness and contrast settings to clearly show localization of tagged proteins. The arrows in the TL panels highlight vacuoles. Yellow arrows indicate endoplasmic reticulum-like localization. 25P indicates Wtf25poison, 25A indicates Wtf25antidote, TL indicates transmitted light, and the scale bar is 4 µm. (TIF) [file pgen.1011534.s014.tif]
