# Supplementary material for: Functional constraints of wtf killer meiotic drivers
Source: PLoS Genet. 2025 Feb 18;21(2):e1011534. doi: 10.1371/journal.pgen.1011534 (PMC11892871; doi:10.1371/journal.pgen.1011534)
Supplement: Attachment — Submitted filename: FinalResponse to reviews.pdf [file pgen.1011534.s023.pdf]

Dear PLOS Genetics editor/reviewers,
We received three constructive reviews of our manuscript from our previous submission to PLOS Biology (tracking number [PBIOLOGY-D-24-02419R1], editor Roland Roberts). We have carefully considered and used each of these reviews to substantially revise our paper to improve the clarity of the experiments and our claims. We also changed spot assays shown in Fig 4B, Fig 4D, S3 Fig G, S4 Fig B, S4 Fig C, S13 Fig B, S14 Fig B-D, to different experimental replicas. While the narrative has changed to better describe how the data support our claims, none of the phenotypes or conclusions have changed from the initial submission.

The reviewers' comments are each presented fully below in black font, although we have broken up some of their paragraphs into shorter segments interspersed with our responses. Our responses are presented in blue font and describe changes we made to the manuscript to address reviewer feedback. We are thankful to the reviewers for their time and efforts and feel our paper is significantly improved from this peer review process. We feel that our current manuscript would be a good fit for PLOS Genetics.

Reviewer #1:

Srinivasa et al. 2024 review

Srinivasa et al. set out to determine the molecular features that define Wtf poison/antidote compatibility and to build on their previous work understanding the mechanisms by which Wtf poison alleles drive toxicity. To me, this paper can be divided into three major parts. Figures 1 and 2 (and supp data) describe clear hypothesis-driven experiments supporting the idea that self-assembly is a prominent (but importantly not sufficient) feature of Wtf poisons. Figures 3 and 4 (and supp data) test clear hypotheses regarding how size and solubility affect Wtf toxicity, but the results are nuanced and difficult to interpret. Figures 5 and 6 (and supp data) feel like the authors were trying a bunch of interesting genetic manipulations and the results were, to me, largely inconclusive.

We thank the reviewer for pointing out our communication failures regarding the experiments in Figures 5 and 6. We have revised the manuscript to better set up the reasons the experiments were conducted, the results and their implications in our revision. Figure 5 (and supp data) tests the hypothesis that the role for Wtf^{poison}/$Wtf^{antidote}$ co-assembly is to physically associate the proteins so that they can be co-trafficked to the vacuole. The experiment tests that idea by artificially tethering (with GBP-mEOS interaction) a poison to an antidote to which it cannot co-assemble. This uncouples physical association from Wtf^{poison}/$Wtf^{antidote}$ co-assembly. Our experiments demonstrate that physical association between Wtf^{poison} and $Wtf^{antidote}$ proteins is insufficient for antidote function (using two distinct Wtf^{poison} and $Wtf^{antidote}$ pairs). This allows us to reject the hypothesis that co-assembly serves only to link a Wtf^{poison} to a functional $Wtf^{antidote}$ and reveals that co-assembly with a $Wtf^{antidote}$ has a broader role in efficient neutralization of a Wtf^{poison}.

We disagree that the results in Figure 6 are inconclusive. This figure, and the associated supplements demonstrate a new type of self-killing wtf allele where the Wtf^{poison} is not efficiently rescued by the corresponding $Wtf^{antidote}$. The existence of such alleles shows that matching amino acid sequence is not always sufficient for Wtf^{poison} and $Wtf^{antidote}$ compatibility. This reveals novel constraints on the evolution of the *wtf* genes and suggests they are bigger contributors to infertility that we previously guessed.

This paper is largely well written, covers a fascinating topic, and includes a ton of data. After reading the introduction and first few paragraphs of the results, I expected to come away with a new understanding of molecular properties that drive protein/antidote biology beyond what was shown in the (outstanding) Nuckolls 2020 paper. This didn't really happen. This paper is great at showing what doesn't drive toxicity and protein/antidote compatibility but less good at clarifying what does.

Thank you for your compliments on our previous work. We agree with the reviewer that we still do not have a complete understanding of these proteins, but we have made substantial progress in identifying factors critical for poison function (self-assembly into small, distributed aggregates), identifying a crucial role for co-assembly in antidote function, and in uncovering the functional implications of the rapidly evolving wtf coding sequence repeats. Our abstract makes the following major claims that are novel to this manuscript. We have significantly revised the paper in an attempt to make these things more clear.

1) Self-assembly is conserved in Wtf^{poison} proteins
Self-assembly was previously demonstrated with Wtf4 in our 2020 paper. Importantly, self-assembly could have been a feature of the Wtf4^{poison} that was present, but not required for toxicity. As not everything that exists is functionally important, we set out to test the idea that the previously observed assembly is functionally important. Showing broad conservation of Wtf^{poison} assembly over 100 million years (despite almost no amino acid conservation), as we have done in this work, provides the first strong support for a functional role of self-assembly in poison toxicity. We also show that toxicity strongly correlates with intracellular Wtf^{poison} localization across many independent alleles: ER-localization is associated with reduced or no toxicity, while distributed aggregates are associated with higher toxicity.

2) Modifying Wtf^{poison} protein assembly with tags modifies its toxicity
This claim complements claim 1 and further supports a functional role of self-assembly in poison toxicity. No previous publications modify Wtf^{poison} assembly properties.

3) Co-assembly of Wtf^{poison} and Wtf^{antidote} promotes effective neutralization of Wtf^{poison} proteins. There are no other published experiments that examine the role of Wtf^{poison}/Wtf^{antidote} co-assembly in antidote function. Our working model was that co-assembly served only to physically link the two proteins to allow co-trafficking. Our new results disprove this idea as physical linkage to a functional antidote, without co-assembly, does not efficiently neutralize Wtf^{poison} proteins.

4) Self-killing *wtf* alleles that encode a functional Wtf^{poison} that is not effectively rescued by a sequence-matched Wtf^{antidote} are possible. Such alleles have not been formally published but they have profound evolutionary implications. Importantly, these alleles can be generated by mutating the *wtf* coding sequence repeats that exhibit extremely rapid evolution. Given the way the *wtf* genes evolve (non-allelic gene conversion) one of the self-killing alleles (repeat deletion) we identified is quite likely to be sporadically sampled in natural populations. This expands the ways *wtf* genes are likely limiting the fitness of populations. In addition, the self-killing alleles reveal future paths of inquiry to understand the requirements for Wtf^{poison}/Wtf^{antidote} compatibility.

I felt that many of the experiments were interesting but inconclusive. I appreciate the challenges here - the goals are ambitious and breaking open the underlying mechanisms of these assemblies is tough. Whether this paper got there or not, I can't say. I really want to like this

paper, but, to me, the results point this way and that, and it was hard for me to keep it all straight. Maybe that just reflects the biology of the system.

We agree with the reviewer that keeping everything straight with this story is a challenge as we generated and tested over 30 alleles. We thank the reviewer for their feedback and have used it to improve our communication. Our changes include renaming our alleles, an additional summary/model figure and substantial rewriting. It is not, however, clear which experiments the reviewer finds inconclusive, beyond figures 5 and 6, which we address above. It is also unclear which experiments the reviewer feels leads to conflicting conclusions.

We suspect the reviewer's perspective that our results are contradictory reflect a communication failure on our part. Our data include one exceptional allele, now called *wtf4*-Δ^{10}, that encodes a Wtf^{poison} protein that assembles into small, distributed aggregates like wild-type, but is surprisingly non-toxic. We do not feel this allele undermines our claims that toxicity is associated with small distributed assemblies, but instead reveals that those things are not all that is required for toxicity, an additional novel discovery of this work. This exceptional allele is valuable, and we intend to use *wtf4*-Δ^{10} to understand what separates toxic from non-toxic assemblies in future work.

We also recognize that the MBP tag had different effects on the Wtf4^{poison} and Wtf25^{poison} proteins. Even so, the observed results were consistent with our claims regarding toxicity. Specifically, the MBP tag reduces the assembly of Wtf4^{poison} proteins, but the aggregates remain distributed in the cell. This is consistent with our claim that small distributed, aggregates are most toxic. With Wtf25^{poison}, the MBP tag changed the intracellular localization of the poison to a more ER-enriched pattern and reduced toxicity. This is consistent with our claim that ER localization is associated with reduced toxicity. (We could not assay if MBP changes the assembly of Wtf25^{poison} because the high toxicity of the wild-type allele prevented us from reliably measuring assembly.)

Major points:

Line 226- what is the conclusion of the results depicted in Figure 3? Line 241- what is the conclusion of the results depicted in Figure 4? Many paragraphs (others being paragraphs ending in line 308, 409, 415) end with an observation and move to the next experiment with no synthesis. In my opinion, this paper would benefit from more 'connective tissue' linking the experiments through statements of conclusions and logic.

We thank the reviewer for this feedback and have added significant connective tissue throughout the results.

This may be indicative: the results are hard (for me) to interpret. Line 398- the authors assayed the effects of deleting the wtf4 exon 6 alone. What's the rationale? By the end of this paper the experiments are being presented rapid-fire without leading the reader through the logic. Maybe this is my problem and it's easier to follow for others, but it was hard to make sense of some of the later experiments/figures.

We endeavored to limit the introduction of each individual mutation to avoid reader fatigue and instead choose to focus on interesting phenotypes. However, the reviewer's comments have helped us recognize that many readers would similarly prefer more explanations, and we have

tried to add additional background and interpretations throughout the results. In particular, we have added an entire new section giving more introduction and discussion to deletion alleles.

Figure 3C does not indicate size of assemblies. Focus size could be a result of multiple closely-spaced assemblies rather than larger assemblies. A biochemical fractionation assay would be useful. Velocity gradient analysis, size exclusion chromatography, or even SDD-AGE would be an important supplement. I understand that analysis by western is tricky for these proteins but is it impossible? Do these proteins not denature when boiled in 2% SDS?

We agree that the size of the assemblies are not reported in figure 3 and we have not claimed to have identified the size at which Wtf^{poison} assemblies are not longer toxic. We agree that such a claim would require some measurement of assembly size. Instead, we have claimed that increasing assembly size suppresses toxicity. The ability of the FTH1 protein to self-assemble is well established (e.g. refs 31 and 32). Our data show an increase in the Wtf^{poison} focus size when linked to FTH1 via the mEos nanobody with five distinct Wtf^{poison} proteins: essentially all the Wtf^{poison} fluorescence is present in one spot. The most parsimonious interpretation of these data is that the FTH1+nanobody forms supermolecular assemblies with the Wtf^{poison}-mEos assemblies.

Alternate models that do not invoke super-molecular assemblies seem improbable. Such models would require either the Wtf or FTH assemblies to be disrupted by the alternate assembly. This disrupted assembly would need to be shared by all five extremely diverse Wtf proteins.

Figure S7 - the Exon 1 when independently expressed cannot function as an antidote. Does it co-assemble with the poison? I think that using co-localization as a proxy for co-assembly (S7G) without the FRET is dicey.

We agree that using co-localization for co-assembly without FRET can be dicey. The specific case which is problematic is if one uses co-localization to prove co-assembly. We have not done this. We have previously demonstrated co-assembly of Wtf4^{poison} with Wtf4^{antidote} using FRET. Because one cannot have co-assembly without co-localization, we have used loss of co-localization of Wtf4^{poison} + Wtf4^{antidote} to infer loss of co-assembly with some Wtf4 mutants. We have also used maintenance of co-localization (often along with re-localization of one protein in the presence of another) as evidence of maintained co-assembly of some type. To clarify, we have added additional explanation throughout the text of our use of colocalization in experiments.

We did not assay if Exon 1 alone can co-assemble with the poison. We anticipate it would not, as there is a high degree of specificity between Wtf^{poison} and their corresponding Wtf^{antidote}s, despite many antidotes sharing rather similar Exon 1 sequences.

Minor points:

Non-toxic Wtf19 that self assembles is interesting. Are there any features specific to this protein that are absent from the other toxic assemblers (or vice versa)?

We agree that Wtf19 is an interesting exception. The only difference we have noted is the enriched ER localization which is also associated with reduced toxicity in several of the mutants we describe. We aspire to follow up with our exceptional mutants in future work to try and understand what determines ER localization.

For Figure 2B (and all additional FRET graphs) the data are too condensed, and I can't see the differences in the distributions. I know you want to capture all the data, but changing the y-axes to zoom in would be nice (maybe eliminating the far tails of the distributions).

Thank you for this suggestion. We have zoomed in on the axes and provided the raw data, including the tails of the distributions in supplemental files.

Figure 2D- authors describe the localization patterns and then claimed that cellular localization (combined with the DAmFRET) is a good but imperfect predictor of toxicity. As written, I don't understand what is predictive about the localization patterns. A synthesis of what patterns are more likely to be toxic might be useful.

We thank the reviewer for pointing this out. We have added additional text to clarify our observations and have added an additional summary figure 7. With the exception of the allele now known as *wtf4*-Δ^{10}, all toxic alleles show distributed assemblies. Besides that exception, mutant alleles that disrupt toxicity (or the wild-type non-toxic allele) have lower DAmFRET, alternate localization patterns (often ER-enrichment), or both.

This is likely out of the scope of the paper but I'm curious - do the assemblies kill or do they inhibit growth? Can you induce synthesis of an assembly and then degrade it?

Unmanaged Wtf^{poison} assemblies kill cells. When we image cells expressing toxic Wtf^{poison} proteins, there is a rather short window of opportunity wherein the cells show fluorescent signal, but are not yet dead (i.e. autofluorescent and/or lysed). This cell death is what limited our ability to reliably assay the very toxic Wtf25^{poison} assembly via DAmFRET, because we need to obtain a sizable population of still viable cells expressing the protein. We have added additional text to make this clear.

We have not, however, determined if pre-assembled Wtf^{poison} assemblies can be rescued by the later induction of a matching Wtf^{antidote}. We are currently working on building tools that will allow us to address this question as part of a future study.

Allelic designations make the paper harder to follow. I'm sure the authors have their reasons but, instead of Wtf4-20(poison), why not Wtf4-NT(poison)? Wtf25-4(poison) could be Wtf25-NT(poison). There are many other examples where the authors could use a more descriptive designation. I appreciate the classical notation but it may come at the cost of readability.

We appreciate this suggestion and have renamed the alleles with more descriptive names.

Line 228 typo: missing an 'of' Wtf (poison)

Thank you, we have corrected this typo.

Reviewer #2:
[identifies himself as Aaron A. Vogan]

Summary:

The manuscript "Functional and evolutionary constraints of wtf killer meiotic drivers" by Nidamangala Srinivasa et al. presents a thorough investigation into the mechanism of toxicity of the wtf spore killer genes from S. pombe. The authors use a large array of molecular techniques to interrogate the hypothesis that aggregation of the wtf-poison is a requirement for toxicity. They conclude that while, indeed, aggregation is important for the function, it is not the sole requirement. I found the manuscript to be in excellent shape and I believe it could be published in its current state. The approaches appear to be quite creative, leveraging a variety of molecular techniques to draw conclusions, and the sections well written.

Thank you for your supportive comments on this work!

One suggestion I have for a change would be to reconsider the title. While I enjoy the discussion on the evolutionary implications of this work, I think it is still fairly speculative without a more detailed understanding of the mechanism of toxicity. As someone who is more of an evolutionary biologist than a molecular biologist, I was expecting a very different paper when I read the current title.

Thank you for this suggestion. We have changed the title to ‘Functional constraints of *wtf* killer meiotic drivers.’

minor points:

Line 72: "D366NN mutation" -> is that changing 2 amino acids, or adding an additional one?

The allele changes swaps one amino acid for two different ones. We have revised the text to clarify.

I have difficulty seeing some the localization of the signals in the microscopy images, is it possible to add more labels or arrows as in S9E? Particularly for the ER localization.

We agree that these localization patterns are not always obvious, particularly when one is presented a representative cell rather than a field (which is impractical). We have provided additional markers on images in figures throughout to highlight localization patterns mentioned in the text.

What's the variation in the ability of the antidote to rescue the cells from toxicity? From looking at the many control experiments with wtf4, it seems that occasionally there is not full rescue, e.g. figure 4C. Does this effect conclusions about the ability of some of the modified alleles to act as antidotes?

Good observation and question. With the inducible system used in this paper, we can almost always see less growth of cells expressing the $Wtf4^{poison}$ + $Wtf4^{antidote}$ relative to cells expressing only $Wtf4^{antidote}$ or empty vector. We define this wild-type restoration of growth as full rescue for Wtf4. We also see some variability in our growth assays of this wild-type state. Because of this, we only compare groups of cells that are prepared in parallel and grown on the same plate. If cells are within a figure panel, they were grown on the same (usually large) plate and the white gaps are due to moving the dilutions that were not adjacent on the plate. To claim deviations from the wild-type level of rescue, we test multiple transformants (usually from multiple plasmids) for each genotype and require that the observed growth differences (relative to the wild-type on the same plate) are repeatedly observed.

While it is too much to add to the supplemental data provided in the publication, all the raw data (including independent replicates) of all the experiments presented in this paper will be available in the Stowers online data repository (a mandate at our institution).

Reviewer #3:

In this manuscript, Nidamangala Srinivasa and colleagues explore the mechanisms of toxicity and rescue of the wtf meiotic drivers in yeast. While previous studies, particularly on the Wtf4 driver, have explored these mechanisms, this work focuses on the natural diversity of wtf drivers across distant yeast species to identify principles that underpin the evolution of their toxicity and rescue functions.

I find the wtf drive system fascinating, and there remains much to uncover about the mechanisms involved. However, I struggled to extract a clear take-home message from this manuscript that distinguishes it from what is already established in the literature... This may be due, in part, to the manuscript feeling more like a patchwork of various experiments rather than following a cohesive narrative. Key ideas or findings might have been overlooked as a result. This highlights the need, in my opinion, for the authors to reconsider the central message they wish to convey and do it more efficiently. Currently, the narrative seems to follow a "we tried this, we tried that" flow, with many experiments yielding inconsistent results between drivers, leading to the main conclusion that these drivers are highly "idiosyncratic". While this could be true, it more likely reflects our still limited understanding of the system and the constraints of the methodologies used. In all, there are interesting insights/ideas here, but these are not easily accessible.

We appreciate the reviewer’s time and feedback and we have extensively revised the paper to make our main conclusions more accessible.

Major points

1. While molecular genetic approaches to dissect the mechanism of proteins are extremely powerful, I think that the experiments performed in this study have important limitations, especially when it comes to exploring complex biochemical processes such as oligomerization and self-assembly of both toxins and antidotes. Such molecular genetic approaches: including fusions of toxins and antidotes to fluorescent proteins (for co-localization), FTH1 (for altering assembly size), spidroin/MBP (for altering solubility), GFP/GBP tethering (for protein interaction studies), and domain deletions are most informative when there is already some structural understanding of the proteins involved. This foundation allows for drawing stronger conclusions and better control over experimental variables. Any of these protein fusions could introduce artifacts, and the manuscript provides several examples where tagging clearly impacts protein function, raising concerns about the overall interpretability of the results.

We agree that a structural understanding of Wtf protein assemblies would be valuable. We are currently trying to fill this gap, but it is challenging. We do not have a structure of any Wtf protein and obtaining such a structure would be outside the scope of this paper. Structural prediction programs also currently offer limited insight into Wtf protein structures. For example, the alpha fold predictions are low confidence. In addition, the assembly predictions made by alphafold (e.g. between a poison and an antidote) appear faulty as regions we know empirically to be important for co-assembly are not predicted to be important.

Still, we disagree about how much structural understanding is required to conduct strong, rigorously controlled experiments on proteins of any type, including those that assemble. For example, there is minimal understanding of the assembly of unstructured proteins within condensates, but exciting insights about such proteins are published every day.

There are almost no perfect experimental tools, including fusion proteins, which we acknowledge can introduce artifacts. But well controlled experiments, like ours, allow one to use imperfect tools to gain insights. Using tagged proteins to assay colocalization is foundational in cell biology. While fluorescent tags can disrupt Wtf protein function, the wild-type tagged proteins we have presented are functional, as demonstrated by the data in our paper. We then only compare mutant alleles to those tag-matched wild-type controls.

We acknowledge that the spiroidin, MBP and FTH1 tags altered protein function, but that was the intent of those experiments. Our hypothesis was that assembly properties affected protein toxicity, and those tags (designed and characterized by others for their ability to affect assembly properties of proteins of interest) allowed us to test our hypothesis. We used multiple distinct types of assembly modifying tags on multiple dramatically diverse proteins intentionally to mitigate the possibility that our experimental results reflect artifacts. Multiple distinct tags, on multiple distinct proteins all artifactually pointing to the same conclusion (assembly properties affect toxicity) has a low likelihood.

Our tethering experiments in Figure 5, for example, include controls that show that the $Wtf4^{poison}$-GBP-mCh protein and the $Wtf61^{antidote}$-GFP protein are both functional. In addition, we have controls showing that antidote rescue can happen in the presence of the GBP-GFP interaction ($Wtf4^{poison}$-GBP-mCh+ $Wtf4^{antidote}$-GFP). Beyond that experiment, we show the same result using an independent pair of extremely diverged Wtf proteins, along with all the of the same controls described above.

Some experiments also appear overly simplistic. For example, in one case, the N-terminal domain of the Wtf4 antidote was removed and inserted in the "middle of the protein" (line 300), resulting in a non-functional antidote (Wtf4-6). From this, the authors conclude that "shared amino acid identity at the C-terminus is insufficient for poison and antidote co-assembly." This reasoning seems flawed. Ultimately, this is a biochemical problem involving two proteins physically interacting, and as such, relocating a domain arbitrarily to the "middle of the protein" (I apologize if I missed this, but I could not find any more information about this) without any justification based on their protein domain architecture, makes it unsurprising that such an approach disrupts the protein's function. I'm sorry if I sound overly critical but I just think that for the specific questions being asked (formation of self-assemblies and toxin-antidote physical interactions), the proposed experiments are not very informative or insightful.

We appreciate that we did not sufficiently explain our motivation for building each allele. We have included more of such explanations in the revision, but it is still limited as we suspect readers will not have the fortitude to get the full backstory on over 30 alleles. In particular, we rewrote the section describing the wtf4-6 allele and included more details. Our motivation for constructing that wtf4-6 (renamed $wtf4^{poison}$-$ex1^{int}$) allele is layered. 1) There is almost no conservation within Wtf proteins. Moreover, some contain large regions (coding sequence repeats) that are lacking in others. We also knew from our previous work that disruptions of shared amino acid identity at the C-terminus can affect poison/antidote compatibility, whereas some mismatches outside of that region could be tolerated. This suggested that the C-terminal region might constitute a self-assembling domain that must match between poison and antidote

proteins. If this was the case, such a protein could potentially tolerate rather dramatic mutations outside of this region.

2) We wanted to test the modularity of the antidote-specific domain. We found that moving the domain within the protein did not, in fact, totally break the protein. The Wtf4-6^{andtidote} was non-toxic and was efficiently trafficked to the vacuole. Where the protein failed, was with rescue of the poison proteins, which has allowed us to hone our understanding of what is required for poison/antidote compatibility (i.e. the C-terminus is insufficient). We are also interested in using Wtf proteins to understand what is required for assembly/aggregate toxicity in general, and the alleles with exon 1 moved within the center of the protein have proven to be important in those endeavors.

On a positive note, the experiments involving GFP/GBP tethering and the specific deletions of repeat sequences within the C-terminal domain (Figure 6) are particularly intriguing and more straightforward to interpret. I would suggest dedicating more time to explaining, exploring, and refining these experiments, while considering the removal of others, such as those involving FTH1 and spidroin/MBP, which seem too premature or inconclusive at this stage. Less is more.

As described above, we prefer to retain these experiments as they reinforce that our findings are robust in that they are not specific to a given Wtf protein or tag.

2. Nomenclature and clarity of the text and figures. The nomenclature used by the authors to designate a wide range of mutant, truncations, fusions alleles could be drastically improved to facilitate the reading of the manuscript. For instance, Wtf4-20(poison) designates an allele where Wft-4 is fused on its N-terminus to the "mutated N-terminal domain (D40K, K65D) from the flagelliform spidroin 1A variant 1 from Trichonephila clavipes, NT*". So perhaps calling this variant something like "Spi-Wtf4", would be far easier on the reader than trying to remember what exactly is Wtf4-20, and how is it different from Wtf4-28 or Wtf4-13, which as far as I can tell are just arbitrary number designations. Similar with truncations, Wtf4-13 could be designated Wtf4:Δex4 or even better, mention the precise amino acid residue range that is being removed from the protein.

We thank the reviewer for this suggestion and have renamed the alleles.
